# L-Arginine Improves Cognitive Impairment in Hypertensive Frail Older Adults

**DOI:** 10.3389/fcvm.2022.868521

**Published:** 2022-04-12

**Authors:** Pasquale Mone, Antonella Pansini, Stanislovas S. Jankauskas, Fahimeh Varzideh, Urna Kansakar, Angela Lombardi, Valentina Trimarco, Salvatore Frullone, Gaetano Santulli

**Affiliations:** ^1^Department of Medicine, Albert Einstein College of Medicine, New York, NY, United States; ^2^Azienda Sanitaria Locale (ASL) Avellino, Avellino, Italy; ^3^Campania University, Naples, Italy; ^4^University of Naples “Federico II”, Naples, Italy

**Keywords:** cardiac rehabilitation, endothelial (dys)function, L-Arg, L-Arginine, frail adults, frailty, cognitive impairment

## Abstract

Cognitive impairment is a prevailing event in hypertensive patients and in frail older adults. Endothelial dysfunction has been shown to underlie both hypertension and cognitive dysfunction. Our hypothesis is that L-Arginine, which is known to ameliorate endothelial dysfunction, could counteract cognitive impairment in a high-risk population of hypertensive frail older adults. We designed a clinical trial to verify the effects of 4-weeks oral supplementation of L-Arginine on global cognitive function of hypertensive frail older patients. The study was successfully completed by 35 frail hypertensive elderly patients assigned to L-Arginine and 37 assigned to placebo. At follow-up, we found a significant difference in the Montreal Cognitive Assessment (MoCA) test score between the L-Arginine treated group and placebo (*p*: 0.0178). Moreover, we demonstrated that L-Arginine significantly attenuates Angiotensin II-induced mitochondrial oxidative stress in human endothelial cells. In conclusion, our findings indicate for the first time that oral L-Arginine supplementation significantly improves cognitive impairment in frail hypertensive older adults.

**Clinical Trial Registration:**

www.ClinicalTrials.gov, identifier: NCT04962841.

## Background

Hypertension is linked to endothelial dysfunction contributing to atherosclerosis, inflammation, and oxidative stress in the arterial wall (1–8). Physical frailty (hereafter defined as frailty) is a biological syndrome of decreased physiological reserves with increased susceptibility to stressors; stressors are classified as acute or chronic diseases that lead frail patients to mortality, hospitalization, disability, functional and cognitive impairment (9–15). In this scenario, hypertension is considered a common stressor; additionally, frail older adults display endothelial dysfunction as a consequence of the aging process; therefore, frailty and hypertension synergistically increase the risk of adverse events (16–18).

Cognitive impairment is often observed in hypertensive patients as well as in frail older adults (19–21). Hence, tackling cognitive impairment is crucial in order to delay and/or prevent adverse events, complications, and death. We and others have demonstrated that endothelial dysfunction is present in patients with hypertension and cognitive impairment (6, 22–25).

L-Arginine is an amino acid involved in a number of biological processes and is a substrate of two enzymes: nitric oxide (NO) synthase (NOS) and arginase (NOA) (26–28). L-Arginine is fundamental for NO production by endothelial cells, regulating vascular tone and cardiovascular homeostasis (28–32). We hypothesized that L-Arginine could counteract cognitive impairment in a high-risk population such as frail older adults with hypertension. To test this hypothesis, we designed a study to investigate the effects of 4-weeks supplementation of L-Arginine on global cognitive function in hypertensive frail older adults.

## Methods

We designed a placebo-controlled clinical trial to study hypertensive frail older patients presenting from March 2021 to October 2021 at ASL (local health unit of the Italian Ministry of Health) Avellino, Italy. All of them met the following inclusion criteria: a previous diagnosis of primary hypertension (with no clinical or laboratory evidence of secondary causes); age >65 years; a frailty status; Montreal Cognitive Assessment (MoCA) test score <26.

Exclusion Criteria were: age <65 years; presence of neurodegenerative diseases; absence of frailty status; absence of hypertension; left ventricular ejection fraction <25%, with previous myocardial infarction or previous coronary revascularization.

Patients were randomly assigned to the L-Arginine (Bioarginina^®^, 1.66 g, twice a day) or placebo (*n* = 37) parallel groups and followed-up for 4-weeks. The dose of L-Arginine was based on previously published clinical trials (33, 34).

### Assessment of Cognitive Function

Global cognitive function was assessed using the MoCA test, with scores ranging from 0 to 30 (lower scores indicate cognitive impairment), as we previously described (35); this cognitive test covers the main cognitive areas: immediate and delayed memory (free and cued recall), language, visuospatial and visuoperceptual capacities, motor planning, executive function, attention, and cognitive judgment (36–38).

### Frailty Evaluation

A physical frailty assessment was performed following the Fried Criteria (11) as we previously described (35); a diagnosis of frailty status was made with at least three points out of the following five:

- Weight loss (unintentional loss of ≥4.5 kg in the past year).
- Weakness (handgrip strength).
- Exhaustion (poor endurance and energy, self-reported).
- Slowness (walking speed under the lowest quintile).
- Low physical activity level (lowest quintile of kilocalories of physical activity during the past week).

### Cell Culture and Mitochondrial Reactive Oxygen Species (ROS) Detection

Human umbilical vein endothelial cells (HUVECs) were cultured in EGM-2 medium (Lonza, CC4147) and incubated at 37°C and 5% CO_2_ (39). Experiments on HUVECs were performed at passages 3–7. After reaching a 60–70% confluency, HUVECs were plated on glass bottom culture dishes and treated with Angiotensin II (Ang II) (Merck, 05-23-0101, 1 μM) and Ang II with L-Arginine (Fisher BioReagents, BP372-100, 500 μM) in EGM-2 medium for 24 h. ROS generation was quantified using MitoSOX™ Red (Molecular Probes Inc, M36008), incubating cells for 10 min at 37°C and 5% CO_2_, as we previously described (6, 40).

### Study Approval

Informed consent was obtained by all patients before testing, and the experimental protocol was approved by the Ethical Committee of Campania Nord. The trial was registered in clinicaltrials.gov (NCT04962841).

### Statistical Analysis

Data are presented as group mean ± SD or SE or numbers and percentages. Based on our preliminary findings, we calculated the minimum number of patients required for the study to reject the null hypothesis 95% of the time using G^*^POWER. A multivariable linear regression analysis, while adjusting for likely confounders, was used to investigate the association between L-Arginine treatment and MoCA score. Statistical significance was determined by a *p* value <0.05. All calculations were performed using SPSS 26 (IBM, Armonk, NY).

## Results

### Baseline Characteristics of Our Study Population

**Figure 1:**
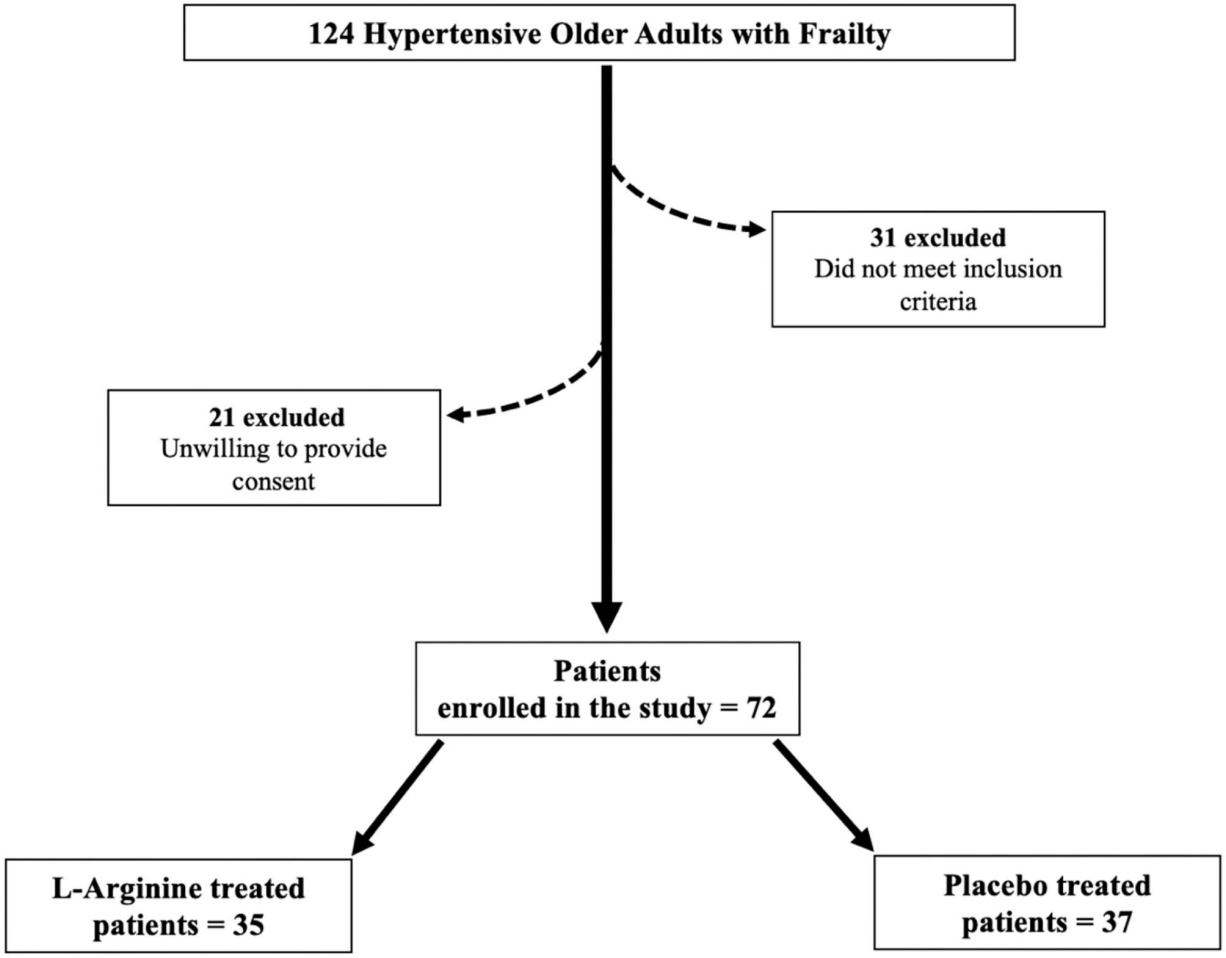
Flow chart of the study.

Seventy two frail hypertensive elderly patients, randomly assigned to the L-Arginine (*n* = 35) or placebo (*n* = 37) parallel groups, successfully completed the study (Figure 1). Their anthropometric features are described in Table 1. There were no significant differences in the mean age, BMI, and sex distribution between the two groups (Table 1). The use of diuretics, angiotensin-converting enzyme inhibitors, beta-blockers, and calcium-channel blockers was similar between the two groups (Table 1).

**Table 1:** Baseline clinical characteristics of our population.

**Parameter**	**L-Arginine**	**Placebo**
*N*	35	37
Age (years)	78.0 ± 6.6	77.0 ± 4.9
Female sex, *n* (%)	21 (60)	23 (62.1)
BMI (kg/m^2^)	29.1 ± 3.4	29.3 ± 3.6
SBP (mmHg)	132.8 ± 10.9	129.2 ± 11.8
DBP (mmHg)	82.0 ± 9.4	79.2 ± 6.3
Heart rate (bpm)	73.7 ± 9.1	73.9 ± 8.9
**Global cognitive evaluation**			
MoCA	18.8 ± 3.8	18.97 ± 4.0
**Anti-hypertensive treatments**		
β-blockers, *n* (%)	20 (57.0)	22 (60.0)
ACE inhibitors, *n* (%)	27 (77.0)	29 (78.0)
Angiotensin receptor blockers, *n* (%)	8 (23.0)	9 (24.0)
Calcium channel blockers, *n* (%)	22 (63.0)	25 (66.0)
Diuretics, *n* (%)	11 (32.0)	12 (33.0)
**Laboratory analyses**		
Plasma glucose (mg/dl)	157.1 ± 52.4	154.6 ± 55.3
Creatinine (mg/dl)	1.0 ± 0.2	1.0 ± 0.1
Total Cholesterol (mg/dl)	142.0 ± 14.7	141.8 ± 15.0
LDL Cholesterol (mg/dl)	93.8 ± 8.4	93.4 ± 8.6
HDL Cholesterol (mg/dl)	42.5 ± 5.6	42.2 ± 5.8
Triglycerides (mg/dl)	112.3 ± 6.0	112.7 ± 5.6
**Comorbidities**		
Dyslipidemia (%)	25 (72.0)	27 (73.0)
Diabetes (%)	19 (54.0)	19 (51.0)
COPD (%)	15 (43.0)	17 (46.0)
CKD (%)	16 (46.0)	16 (43.0)
Previous Stroke (%)	5 (15.0)	6 (16.0)
Anemia (%)	8 (23.0)	9 (24.0)
AFib (%)	11 (32.0)	12 (33.0)
**Fried criteria**		
Slowness (%)	27 (77.0)	29 (78.0)
Weakness (%)	28 (80.0)	28 (76.0)
Low Physical Activity (%)	24 (69.0)	22 (60.0)
Exhaustion (%)	10 (29.0)	13 (35.0)
Weight Loss (%)	16 (46.0)	19 (51.0)

*Data are means ± SD or percentages. AFib, Atrial Fibrillation; BMI, body mass index; CKD, chronic kidney disease; COPD, chronic obstructive pulmonary disease; DBP, diastolic blood pressure; HDL, high-density lipoprotein; LDL, low-density lipoprotein; MoCA, Montreal Cognitive Assessment; SBP, systolic blood pressure*.

### MoCA Score at Follow-Up

**Figure 2:**
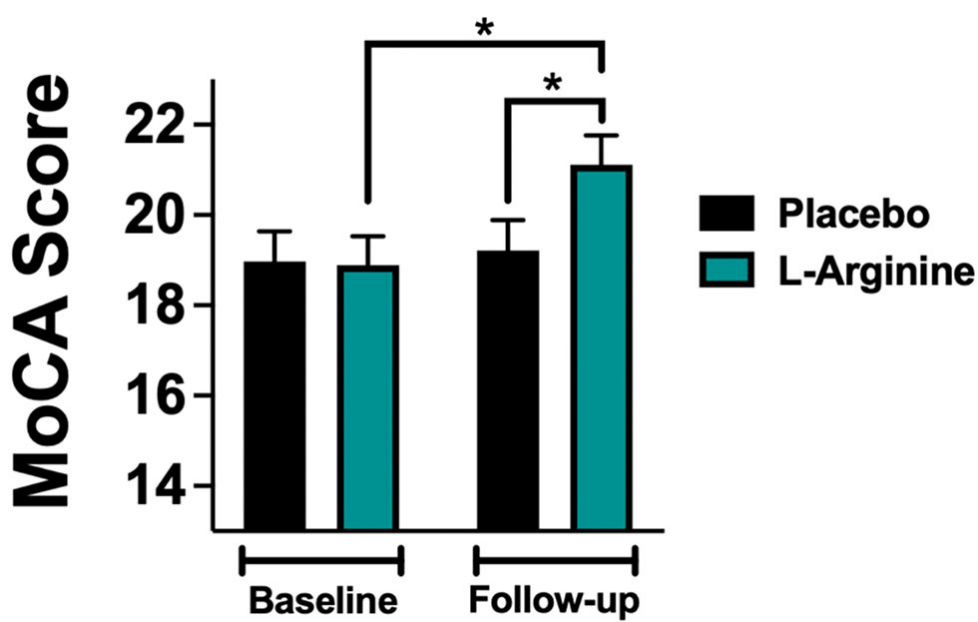
MoCA (Montreal Cognitive Assessment) score evaluated at baseline and at follow-up in the placebo and in the L-Arginine arms. Data are means ± SE; *: *p* < 0.05.

At follow-up, we found a significant difference in the MoCA score between the L-Arginine treated group and placebo (Figure 2). In order to better identify explanatory variables that were associated with MoCA in our trial, we measured the association between L-Arginine treatment and MoCA in a multivariable linear regression model where MoCA was the dependent variable (Table 2). The association between L-Arginine treatment and MoCA score in frail hypertensive patients remained statistically significant after multivariable adjustment for age, BMI, blood glucose, serum creatinine, and blood pressure.

**Table 2:** Multivariable linear regression analysis using the MoCA Score at follow-up as dependent variable.

	***B***	**Standard error**	**Beta**	***t***	***p***	**95.0% Confidence interval**	
						**Lower bound**	**Upper bound**
Age	−0.150	0.069	−0.309	−2.175	0.039	−0.291	−0.008
BMI	−0.199	0.078	−0.180	−2.562	0.016	−0.359	−0.040
Blood Glucose	−0.020	0.012	−0.237	−1.765	0.082	−0.043	−0.003
Serum Creatinine	0.267	1.236	0.016	0.216	0.831	−2.270	2.803
SBP	−0.081	0.043	−0.235	−1.879	0.071	−0.170	0.007
DBP	0.087	0.055	0.233	1.591	0.123	−0.025	0.200
L-Arginine	−2.569	0.837	−0.319	−3.077	0.003	−4.237	−0.900

*BMI, body mass index; DBP, diastolic blood pressure; SBP, systolic blood pressure*.

### L-Arginine Attenuates Endothelial Oxidative Stress

**Figure 3:**
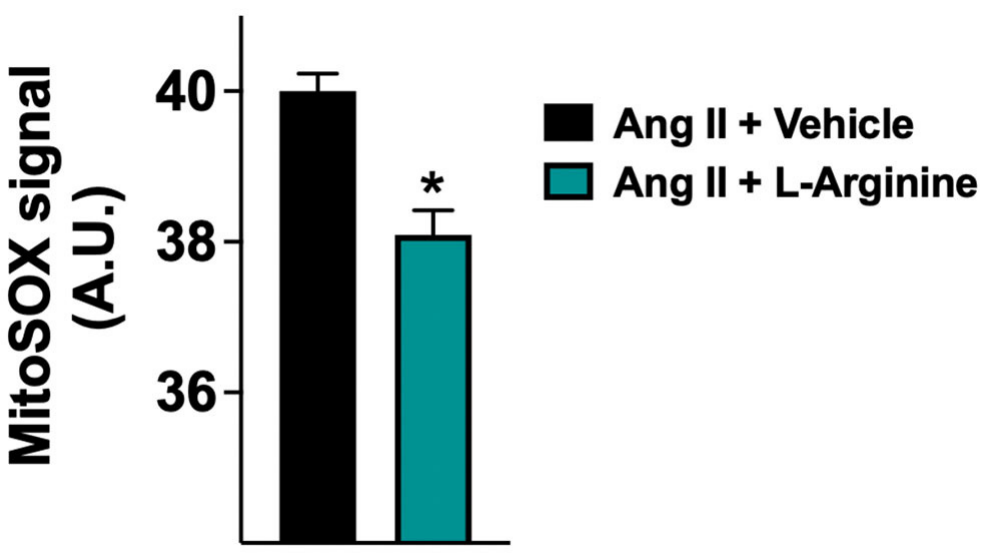
Mitochondrial production of reactive oxygen species (ROS) evaluated in human endothelial cells treated with Angiotensin II (Ang II) (1 μM) + vehicle or Ang II + L-Arginine (500 μM) for 24 hours; A.U., arbitrary units. Data are means ± SE; *: *p* < 0.01.

To provide insights into the physiological mechanisms of protection elicited by L-Arginine, we assessed its effect on human endothelial cells *in vitro*. For this purpose, we used HUVECs, incubated for 24 hours with Ang II (to simulate the hypertensive state) with or without addition of L-Arginine, measuring mitochondrial ROS generation *via* MitoSOX. We found that the co-treatment of HUVECs with Ang II and L-Arginine significantly attenuated mitochondrial ROS production compared to Ang II alone (Figure 3).

## Discussion

The management of frailty in older adults is very debated (41–46). Comorbidities such as hypertension are known to play a key role in increasing the risk of mortality, hospitalization, disability and cognitive impairment. Indeed, hypertension determines alterations of endothelium causing inflammation, atherosclerosis and oxidative stress (47–51). L-Arginine supplementation in elders can equilibrate the L-Arginine/asymmetric dimethylarginine ratio, recovering the production of NO; in fact, the increased L-Arginine availability, resulting from supplementation, competes with asymmetric dimethylarginine in binding NOS (28, 52–54). Furthermore, L-Arginine has anti-inflammatory properties (55–58). In this context, we posited that L-Arginine treatment could improve cognitive impairment in frail older adults for its beneficial action on endothelial dysfunction. We tested this hypothesis in a clinical trial. Our results indicate that adding L-Arginine to standard therapy significantly improves the MoCA Score, indipendently of likely confounders including age, BMI, blood glucose, serum creatinine, and blood pressure.

Global cognitive function was assessed with the MoCA test, which was preferred to the Mini-Mental State Examination (MMSE), because the latter has been shown to be conditioned by demographic variables including age and years of education (59–61). Moreover, the MoCA test is generally considered the best test to detect mild cognitive impairment (62–65).

To mechanistically confirm our results, we examined *in vitro* the effects of L-Arginine on mitochondrial function in human endothelial cells, showing that L-Arginine significantly attenuates the generation of mitochondrial ROS induced by Ang II. Intriguingly, this finding is consistent with recent observations linking frailty and cognitive decline to mitochondrial dysfunction (66–68), which is also a well-recognized determinant of hypertension and vascular aging (69–72).

Several limitations of our study warrant consideration: first, the small sample size; second, we do not know the exact duration of the hypertensive disease; third, the evaluation of the effects of nutraceutical treatment would have benefited from a longer follow-up.

In conclusion, to the best of our knowledge, our study is the first to demonstrate that oral L-Arginine supplementation significantly improves cognitive impairment in hypertensive older adults. Further studies are warranted to verify whether these results can be extended to other populations.

## Data Availability Statement

The original contributions presented in the study are included in the article/supplementary material, further inquiries can be directed to the corresponding author/s.

## Ethics Statement

Ethical approval was obtained from the Ethics Committee of Campania Nord. The patients/participants or their legal representatives provided a written informed consent to participate in this study.

## Author Contributions

PM and GS designed the study, drafted the manuscript, approved its final version, and made the decision to submit and publish the manuscript. FV, UK, VT, and SJ revised the manuscript's intellectual content and approved the final version. AL, AP, and SF acquired the data, revised the manuscript's intellectual content, and approved the final version. PM is the guarantor of this work and, as such, had full access to all the data in the study and takes full responsibility for the integrity of the data and the accuracy of the data analysis. All authors have read and approved this submission.

## Funding

The Santulli's Laboratory is supported in part by the National Institutes of Health (NIH): National Institute of Diabetes and Digestive and Kidney Diseases (NIDDK: R01-DK123259 and R01-DK033823), National Heart, Lung, and Blood Institute (NHLBI: R01-HL146691 and T32-HL144456), National Institute on Aging (NIA: R56-AG066431) to GS, by the Irma T. Hirschl and Monique Weill-Caulier Trusts (to GS), and by the Diabetes Action Research and Education Foundation (to GS). SJ and FV hold postdoctoral fellowships from the American Heart Association (AHA-22POST915561 and AHA-21POST836407, respectively). Both placebo and L-Arginine (Bioarginina^®^) were kindly provided by Farmaceutici Damor S.p.A., Naples, Italy, which had no role in the design and conduct of the study, collection, management, analysis, and interpretation of the data, preparation, review, or approval of the manuscript and decision to submit the manuscript for publication.

## Conflict of Interest

The authors declare that the research was conducted in the absence of any commercial or financial relationships that could be construed as a potential conflict of interest.

## Publisher's Note

All claims expressed in this article are solely those of the authors and do not necessarily represent those of their affiliated organizations, or those of the publisher, the editors and the reviewers. Any product that may be evaluated in this article, or claim that may be made by its manufacturer, is not guaranteed or endorsed by the publisher.
